# Preventive measures for health workers exposed to COVID-19 (SARS-CoV-2)

**DOI:** 10.47626/1679-4435-2022-781

**Published:** 2022-03-30

**Authors:** Jonh Astete-Cornejo, Miguel Burgos-Flores, Kevin Jesús Mayma-Aguirre

**Affiliations:** 1 Universidad Peruana Cayetano Heredia, Unidad de Medicina Ocupacional y Medio Ambiente, Lima (Lima), Peru.; 2 Instituto Nacional de Salud, Centro Nacional de Salud Ocupacional y Protección del Ambiente para la Salud, Lima (Lima), Peru.

**Keywords:** COVID 19, occupational risk, health worker, occupational health, prevention and control

## Abstract

COVID-19 is a disease caused by a new coronavirus that presented an epidemic focus in China in December 2019 and was declared as a pandemic months later. Consequently, the health systems of most countries implemented preventive measures for their population, thus affecting health personnel, which is the first response force. According to the World Health Organization, 37 million health workers fell ill with COVID-19. In this article, we seek to identify strategies for the prevention of contagion of health personnel by COVID-19 that have obtained favorable results and present measures applicable to the Peruvian reality, focused on the personnel that make up the diagnostic process of COVID-19 and the health centers in operation during the health emergency due to COVID-19. It is concluded that temporary confinement in rotating days of health personnel, traffic control bundling, and adequate supply of personal protective equipment were those that have favored the lower incidence of cases of contagion in health personnel in the countries where they were used.

## Introduction

On March 6th, 2020, the President of Peru announced the first case of coronavirus infection, COVID-19, in the country.^1^ According to the Epidemiological Alert no. 011-2020, the first case was a Peruvian citizen who had travelled to Spain, France, and the Czech Republic; on March 12^th^ 22 cases were confirmed, including imported ones and their direct contacts.^2^

In view of this scenario, there was the implementation of measures such as epidemiological surveillance, encompassing from the search for suspected cases of direct contact transmission to household isolation of confirmed cases; laboratory procedures to confirm the diagnosis of COVID-19; basic measures to prevent and control contagion in health centers; clinical management of positive cases and their reporting for epidemiologic investigation.^2^

Given these circumstances, the President of Peru, through Supreme Decree no. 008-2020-SA, declared the state of national health emergency for 90 days to prevent and control COVID-19 contagion, establishing measures that included strengthening of the health system and limited social interactions in the national territory.^3^ Subsequently, a state of emergency was declared for 15 calendar days as of March 16th, with more severe measures such as mandatory social isolation and closure of borders,^4^ which was later extended up to May 2020, with mobility restrictions; subsequently, there was the resumption of economic activities.^5^

One year later, on March 7th, 2021, Peru had 1,371,176 confirmed cases, with the highest rates being observed in the regions of Lima (591,953), Arequipa (61,832) Callao (60,235), Piura (51,093), and La Libertad (47,096), in addition to 47,854 deaths.^6^

With regard to health workers, most health care professionals are in direct contact with patients with confirmed or suspected COVID-19 or with their biological sample; thus, these professionals are at higher risk of contagion.^7^

In countries like Italy, Spain, China, where reports show that the mortality rates due to this coronavirus are currently of 10.08%, 7.27% and 4.02%, respectively, preventive measures have been taken for the general population and for health personnel.^8^ The World Health Organization (WHO) reports that, up to February 2nd, 2021, 37 million of COVID-19 cases were notified in health workers of 183 countries and territories, of which 68% were women, accounting for 36% of the total number of cases worldwide.^9^

In view of this information and knowing that health personnel are in direct contact with patients with suspected or confirmed COVID-19, as well as with their biological samples, it is necessary to take measures to prevent contagion among health personnel in our countries.^10^

## International context

In countries where mortality rates are low, such as Germany and South Korea, five measures to prevent massive contagion of COVID-19 stand out.^8,10^

1. A great number of tests were performed for early detection of people infected with the virus.
2. Isolation of infected patients. In South Korea, all individuals with fever were assessed to rule out COVID-19, and positive cases were isolated in the so-called quarantine hotels. Taiwan, in turn, performed clinical and laboratory tests in all individuals coming from Wuhan, the city where the outbreak started.
3. Rapid preparedness and reaction, through which Taiwan and Singapore were able to contain massive spread by detecting and isolating new cases of COVID-19. Meanwhile, Hong Kong implemented temperature monitoring stations in its ports of entry and isolation of 14 days for tourists arriving in the country.
4. Social distancing, since promptness in implementing social distancing regulations in countries such as Hong Kong and Taiwan was crucial to reduce contagion, with the suspension of school classes and social events. Conversely, Singapore decided to implement measures such as temperature monitoring of all students and the teaching staff every class day.
5. Hygiene measures. In Singapore, Hong Kong and Taiwan, antibacterial gel dispenser stands are common in the streets, in addition to national campaigns to promote hand washing and respiratory hygiene.

With regard to Singapore, the lessons learnt in 2003 with SARS, which infected 41% of health personnel in the country, led the Ministry of Health of this country to implement measures to protect its health professionals.^11,12^

Measures consisted of implementing the Systems Engineering Initiative for Patient Safety (SEIPS) model, which is centered on workers’ safety and is supported by four factors to ensure safety and implementation of the model:

1. Health care tasks: Work tasks should be designed by separating the health team that provides care to cases of suspected and confirmed COVID-19 from those who handle other patients. This minimizes the risk of cross-infection among patients and health workers and determines the appropriate personal protective equipment (PPE) for each professional according to occupational exposure.
2. Tools and technologies: Instruments are used to diagnose COVID-19 in individuals working in the frontline. Since the result of these instruments is not as fast as that of other laboratory tests, suspected cases are isolated in a different area from that of emergency patients. Furthermore, workers’ temperature is monitored twice a day and registered in a government system that monitors variations in measurements for each worker, including when they are not in their workplace.
3. Physical and environmental factors: This aspect involves monitoring the location of health professionals, thus prohibiting them to work in more than one public or private health center. The health staff in each center is reduced to the minimum necessary to prevent intra-hospital infection and to prevent the increase in the number of health professionals in quarantine. The lunch period is divided into different times and groups, meetings are held by videoconference, and entrance of students and health practitioners is prohibited.
4. Organizational conditions: They consist of efficient management of both personnel and PPE. Following the crisis caused by the severe acute respiratory syndrome coronavirus 2 (SARS-CoV-2), the government established a program of PPE storage in view of a possible pandemic; therefore, all health care professionals are duly protected. Patients are separated according to health groups by levels of exposure. Information on prevention is daily sent by mail.

**Figure 1:**
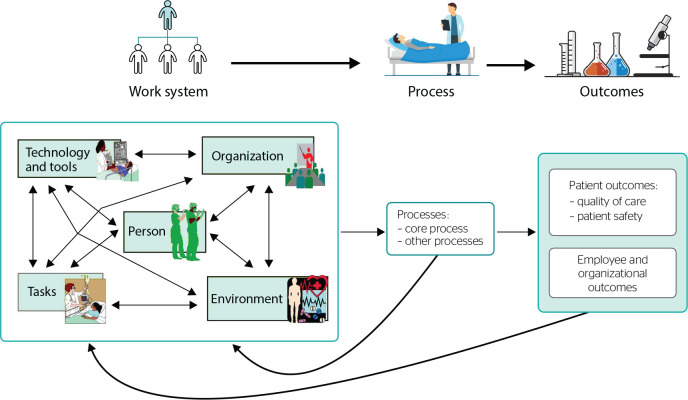
Structure of the SEIPS model.

Source: Carayon et al.^13^

Moreover, it is indicated that, in a public health crisis, health workers not only have to work increased hours, but also often work in a context in which knowledge and understanding on the new pathogen are not still optimal. Donning and doffing the entire PPE are added to physical fatigue and psychological stress; therefore, it is important to emphasize the role positive leadership and support to health care professionals as parts of the SEIPS model^11,12^ (Figure 1).

## National context

In Peru, the first imported case of COVID-19 was confirmed on March 6th, 2020. However, contention measures for the isolation of this person and his contacts took a long time to be applied and were not appropriately monitored; therefore, other cases were confirmed among his contacts. Currently, the country is in a national health emergency, in which mandatory social isolation was implemented to prevent spread of virus and to prevent cases that could be severe and require hospitalization.^13^

However, with regard to preventive measures that should be taken by health professionals who have contact with patients with confirmed or suspected COVID-19, it is worth considering whether these professionals need to be confined in their workplace.

## Objective

To preserve the health of both health care and non-health care professionals who perform activities that involve direct contact with patients, biological samples, and biological waste of cases of COVID-19.

To report the confinement measures that were taken in other countries and their effectiveness.

## Preventive measures among health professionals

It is necessary that all health professionals adopt biosafety measures that include the use of mandatory PPE and good practices for its use and disposal,^14^ in addition to hygiene measures consisting of hand washing and disinfection of all material handled by health personnel inside the health center^14,15^ (Table 1).

**Table 1:** Use of personal protection according to health care level

Type of care	Hand hygiene	Disposable aprons	Medical mask	N95 or FFP2	Protection goggles or face shield	Latex or nitrile gloves
Triage	×		×			
Collection of samples for laboratory diagnosis	×	×		×	×	×
Case of suspected or confirmed COVID-19 requiring admission to the health facility without AGP	×	×	×		×	×
Case of suspected or confirmed COVID-19 requiring admission to the health facility and AGP	×	×		×	×	×

AGP = aerosol-generating procedures

Source: Pan American Health Organization.^15^

Social insurance in Peru reports that the highest percentage of infected health workers was physicians, including residents and surgeons. In the systematic review conducted by the Pan American Health Organization (PAHO), SARS-CoV-2 infections occurred among health workers both clinical and non-clinical areas, there were no consistent differences in the risk of infection according to work positions, and no association was found between sex or age and risk of SARS-CoV-2 infection or seropositivity.^9,16^

When a health professional is infected, it implies their mandatory isolation, as well as isolating individuals with whom they had contact in the health facility and performing tests to detect the virus in all of their contacts. However, health professionals who have high-risk contacts because they work in a hospital or health center may serve as a route of transmission to their relatives, despite not being infected; thus, some countries implemented confinement of health professionals in their workplace.^17^

This confinement is understood as providing an environment for them to rest and/or live while working with risk of exposure to COVID-19. Health professionals who get infected cannot remain confined together with those who are not infected. However, the PAHO only proposes methods of isolation in suspected and confirmed cases, following isolation conditions to respond to COVID-19 in hospitals.^18^

One of the recommendations to significantly reduce COVID-19 infections among health personnel and patients was suggested soon after Taiwan implemented a Traffic Control Bundling (TCB), to prevent contagion among health personnel and patients with SARS, whose guidelines are:

1. To perform triage outside hospitals, in field tents, ensuring that patients who tested positive are sent to an isolation zone with individual isolation rooms where they will subsequently be treated.
2. Patients presenting with inconclusive symptoms or undetermined laboratory tests will need to be sent to a quarantine room, where they will stay during the incubation period.
3. These zones should be clearly distinguished, with a route different from that used by health professionals who circulate through the medical center.

The efficacy of the TCB in preventing SARS was confirmed in Taiwan, where there were only two cases of infected health workers in the 18 hospitals where the TCB was implemented, whereas there were 115 health workers infected with SARS in the 33 hospitals without this system.^19,20^

In China, more than 3,000 health professionals were infected with COVID-19, a fact that may be related to the exhaust air volume in ventilation systems for hospitals, which was approximately 150 m^3^/h per person, much lower than the guideline of 288 m^3^/h per person advised by the WHO for infection control in health care for natural or mechanical ventilation systems.^9,21^

Many health workers present with factors that increase their risk for severe infection or death from COVID-19; thus, organizations should decide whether these workers, including physicians, should be redistributed outside places of greater risk. Although risk cannot be completely eliminated, some sensible adjustments are indeed justifiable; a solution could be places requiring the experience of physicians and nurses with telemedicine services to refer suspected patients.^21^

## Conclusions

With regard to health workers, their working hours, confinement measures, work environment, biosafety measures, and PPE, the following measures are proposed, based on the information analyzed:

### Collective protection measures

- Appropriate distribution of care zones categorized into high, medium, and low risk of exposure, which should be properly indicated and where only authorized personnel are allowed, using the three guidelines of the TCB implemented in Taiwan.
- Assessment of safety conditions in points of care according to area of risk, environmental factors (temperature, humidity, and air flow); and location factors (distribution, type of floor, cleaning); c) Habitability conditions: resting areas inside health facilities for health personnel, toilets, and changing rooms.
- Adequacy of working schedule 12 hours a day at most for 7 or 10 days of work followed by 14 days of isolation, prior to 7 days of rest at home, for which it is worth considering the assignment of hostels or hotels for isolation that ensure appropriate rest, feeding, and access to communication media for workers; e) Reassignment of roles by workers’ group of risk; f) Establishment of a system of daily self-report of symptoms for health personnel.

### Individual protection measures

#### Preventive measures for health personnel

It is necessary that all health professionals adopt measures of infection control that the use of PPE and good practices for its use and disposal (Table 1),^14^ in addition to hygiene measures consisting of hand washing and disinfection of all material handled by health personnel inside the health center.^4,15^

Furthermore, employers in public and private services should implement vaccination programs against respiratory diseases, including COVID-19. In most countries, the vaccination of the worker population is provided by the government; however, it should be implemented on a continuous basis, assessing the characteristics of immunological response of worker populations. This vaccination brings many benefits both for employers and for workers, since it maintains the workplace with less risk of occupation exposure to COVID-19.^23^

#### Occupational medical surveillance to health personnel of health facilities exposed to COVID-19

- High and medium risk work positions (samplers, laboratory analysts, individuals working in triage, intensive care units, and COVID-19 wards, and cleaning personnel of high-risk areas)
  1. Temperature monitoring (daily)
  2. Respiratory symptoms: self-report (daily).
  3. Serological analysis of exposure to COVID-19 every 7 days.
- Low-risk work positions (administrative and other hospital areas, including the security personnel)
  1. Temperature monitoring (daily)
  2. Respiratory symptoms: self-report (daily).
  3. Serological analysis of exposure to COVID-19 every 14 days.

All workers with reactive results for serological surveillance tests should enter into isolation.

The current situation of uncertainty experienced by health professional during the COVID-19 pandemic, as well as the stress resulting from difficulties in screening, collection of samples, analysis and care to positive COVID-19 patients, and work overload, requires a special psychological monitoring of health personnel. Lack of means to take care of oneself is a psychosocial risk factor for several mental health diseases, namely occupational stress, anxiety, depression, and, in many cases, even burnout.

It is important to conclude with the administrative approach, since all these measures could come into effect through the favorable opinion of the corresponding government entities and through the organization of systems within each institution, especially those of human resources and procurement.

## References

[1] El Peruano (2020). Presidente pide a la población mantener la calma al confirmar primer
caso de coronavirus en el Perú [Internet].

[2] Perú, Ministerio de Salud (2020). Alerta epidemiológica codigo: AE-011-2020 [Internet].

[3] El Peruano (2020). Decreto Supremo que declara en Emergencia Sanitaria a nivel nacional por
el plazo de noventa (90) días calendario y dicta medidas de prevención y
control del COVID-19 [Internet].

[4] El Peruano (2020). Decreto Supremo que declara Estado de Emergencia Nacional por las graves
circunstancias que afectan la vida de la Nación a consecuencia del brote del
COVID-19 [Internet].

[5] El Peruano (2020). Decreto Supremo que prorroga el Estado de Emergencia Nacional por las
graves circunstancias que afectan la vida de la nación a consecuencia del
COVID-19 y dicta otras medidas [Internet].

[6] Perú (2020). Sala situacional COVID-19 [Internet].

[7] Gan WH, Lim JW, Koh D. (2020). Preventing intra-hospital infection and transmission of
coronavirus disease 2019 in health-care workers. Saf Health Work.

[8] Johns Hopkins University (2020). COVID-19 dashboard [Internet].

[9] Organización Panamericana de la Salud (2021). Actualización epidemiológica enfermedad por coronavirus (COVID-19)
[Internet].

[10] CNA (2020). China says more than 3,000 medical staff infected by COVID-19
[Internet].

[11] BBC News (2020). Coronavirus: 5 estrategias que están funcionando en los países que han
logrado contener los contagios de covid-19 [Internet].

[12] Singapore, Ministry of Health (2003). Special feature: severe acute respiratory syndrome [Internet].

[13] Carayon P, Schoofs Hundt A, Karsh BT, Gurses AP, Alvarado CJ, Smith M (2006). Work system design for patient safety: the SEIPS
model. Qual Saf Health Care.

[14] OSHA (2020). Healthcare Workers and Employers [Internet].

[15] Organización Panamericana de la Salud (2020). Requerimientos para uso de equipos de protección personal (EPP) para el
nuevo coronavirus (2019-nCoV) en establecimientos de salud
[Internet].

[16] Organización Mundial de la Salud (2020). Consejos sobre la utilización de mascarillas en el entorno comunitario,
en la atención domiciliaria y en centros de salud en el contexto del brote
de nuevo coronavirus (2019-nCoV) [Internet].

[17] De La Cruz-Vargas JA. (2020). Protegiendo al personal de la salud en la pandemia
COVID-19. Rev Fac Med Hum.

[18] Figueroa L, Blanco P. (2020). Infección por coronavirus COVID-19 y los trabajadores de la
salud: ¿quién es quién en esta batalla?. Rev Hosp Emilio Ferreyra.

[19] Organización Panamericana de la Salud (2020). Lista de verificación de alistamiento para la respuesta al nCoV 2019 en
hospitales [Internet].

[20] Schwartz J, King CC, Yen MY. (2020). Protecting health care workers during the coronavirus disease
2019 (COVID-19) outbreak - lessons from Taiwan’s SARS
response. Clin Infect Dis.

[21] Chen C, Zhao B. (2020). Makeshift hospitals for COVID-19 patients: where health-care
workers and patients need sufficient ventilation for more
protection. J Hosp Infect.

[22] Adams JG, Walls RM. (2020). Supporting the health care workforce during the COVID-19 global
epidemic. JAMA.

[23] Centers for Disease Control and Prevention (2021). Division of viral diseases (DVD) [Internet].

